# Everyday robotic action: lessons from human action control

**DOI:** 10.3389/fnbot.2014.00013

**Published:** 2014-03-17

**Authors:** Roy de Kleijn, George Kachergis, Bernhard Hommel

**Affiliations:** ^1^Cognitive Psychology Unit, Institute for Psychological Research, Leiden UniversityLeiden, Netherlands; ^2^Leiden Institute for Brain and Cognition, Leiden UniversityLeiden, Netherlands

**Keywords:** complex action, action control, action sequencing, naturalistic action, goal-directed behavior

## Abstract

Robots are increasingly capable of performing everyday human activities such as cooking, cleaning, and doing the laundry. This requires the real-time planning and execution of complex, temporally extended sequential actions under high degrees of uncertainty, which provides many challenges to traditional approaches to robot action control. We argue that important lessons in this respect can be learned from research on human action control. We provide a brief overview of available psychological insights into this issue and focus on four principles that we think could be particularly beneficial for robot control: the integration of symbolic and subsymbolic planning of action sequences, the integration of feedforward and feedback control, the clustering of complex actions into subcomponents, and the contextualization of action-control structures through goal representations.

## INTRODUCTION

In a relatively short time span, the discipline of robotics has advanced from producing industrial non-autonomous, repetitive machines to semi-autonomous agents that should be able to function in a dynamic, human-driven world. Simple examples include automatic vacuum cleaners such as Roombas, but more flexible and autonomous humanoid robots are currently under development (e.g., the RoboHow.Cog project: www.robohow.eu). As robots perform more and more everyday human activities such as household chores, interacting with humans, and thereby almost becoming citizens in our societies, we believe that psychologists can provide relevant knowledge about human behavior that is generalizable to robots.

Like early approaches to artificial intelligence (AI), traditional cognitive psychology considers behavior (of biological or artificial agents) to emerge from discrete series of cognitive operations that take information from the environment (registered by sensory organs or artificial sensors), process this information in more or less complex ways, and eventually manipulate something in the environment as a result of this processing. In psychology, this *discrete, serial processing model *of cognition has been successful in explaining various psychological phenomena, but for one reason or another most research has focused on the early and middle stages of this process, leaving action and motor control far behind. Indeed, psychology as an autonomous science has historically shown an impressive neglect of the study of action and motor control, to the extent that it has even been called the “Cinderella of psychology” (Rosenbaum, 2005).

Fortunately, however, more recent approaches have emphasized the role of action not only as an output function but as a precondition and basic ingredient of human cognition (e.g., Clark, 1997; Hommel et al., 2001; O’Regan and Noe, 2001). These recent approaches have criticized the traditional sequential-stage account of human behavior for analyzing action as a consequence of stimuli. They argue that action is more aptly characterized as people’s means to produce stimuli (desired outcomes), rather than as a means to respond to stimuli (Hommel, 2009). Moreover, actions are more than mere ballistic outputs: they are events that unfold in time and that must be structured in such a way that their outcome satisfies current needs and goals. Consider, for example, the act of tea-making, which consists of a number of components: (1) boiling water, (2) putting a tea bag in a teapot, (3) pouring the boiling water in the teapot, and (4) pouring the tea in one or more cups. Executing these different components in such a way that the intended goal is eventually achieved requires planning. In the following, we will provide a brief overview of available psychological insights into how this planning works in humans, and how these insights might inform the creation of robotic everyday action systems. At the moment, although robot actions mimic human action, the control systems are in fact quite different. We will confine our discussion to four principles that we think could be particularly beneficial for robot control: the integration of symbolic and subsymbolic planning of action sequences, the integration of feedforward and feedback control, the clustering of complex actions into subcomponents, and the contextualization of action-control structures through goal representations.

## INTEGRATING SYMBOLIC AND SUBSYMBOLIC PLANNING

In contrast to the ballistic, single-step actions that participants in laboratory experiments often carry out, everyday action commonly consists of multiple components, as in the tea-making example. In AI and robotics, multi-component actions are commonly planned at a symbolic level, with each action component being represented by an arbitrary symbol or function. The STRIPS (Stanford Research Institute Problem Solver) planner (Fikes and Nilsson, 1971) is a famous example: it serves to translate an initial state into an intended goal state by determining the subset of actions (defined as a symbolically described relation between sets of pre- and post-conditions) needed to do so. The format of all representations involved is symbolic allowing all goals and actions to be represented in basically the same way, although they can be arbitrarily linked to subsymbolic trigger states. This uniformity allows for a very efficient planning process, as action components can be easily manipulated and exchanged until the entire plan is optimal.

Symbolic action planning of this sort is consistent with early models of human action planning, which typically connected underspecified symbolic action representations with subsymbolic trigger states that took care of timing. For instance, Margaret Washburn considered that later action components might be triggered by the perception of the execution of the previous one: “If the necessary stimulus for pronouncing the last syllable of a series were the muscular contractions produced in pronouncing the next to the last syllable, then the proper sequence of movements would be insured” (Washburn, 1916, p. 9). Along the same lines, James (1890) suggested a *serial chaining model*, according to which each action component is triggered by the perception of the sensory feedback produced by the previous component. Accordingly, learners will create associations linking the motor patterns and their sensory consequences in a chain-like fashion.

As more studies were conducted, however, it was found that chaining accounts of sequential behavior cannot account for several empirical observations. In a seminal paper, the neurophysiologist Lashley (1951) pointed out that the serial chaining models of the time were not adequate, because: (1) movements can still be executed if sensory feedback is impaired; (2) some movements are executed too quickly to have time to process feedback from preceding actions, and (3) errors in behavior suggest the presence of predetermined action plans (Rosenbaum et al., 2007). Rosenbaum et al. (2007) added further arguments against a chaining account of sequential action. For example, the time needed to initiate an action is a function of its complexity (Henry and Rogers, 1960; Klapp, 1977; Rosenbaum, 1987), suggesting that the agent anticipates later action components before beginning to execute the first.

Along the same lines, Cohen and Rosenbaum (2004); [for another good example see Van der Wel and Rosenbaum (2007)] had participants grasp a vertical cylinder placed on a platform and move it to another platform that was either higher or lower than the initial location. The researchers determined the vertical location of the grasp, and found that the grasp location was dependent on the expected end state. More specifically, subjects tended to choose a lower grasp location when bringing the cylinder to a higher position, and vice versa. Likewise, when subjects were asked to move the cylinder back to its starting position, they tended to grasp it in the location where they grasped it before. This *end-state comfort effect* suggests that people anticipate the position that they will assume after the action has been completed.

The same conclusion is suggested by studies on context effects in speech production. For example, people round their lips before pronouncing the *t* in the word *tulip*, in anticipation of pronouncing the *u* later in the sequence (Daniloff and Moll, 1968; Bell-Berti and Harris, 1979; Fowler, 1980; Rosenbaum, 1991). This does not seem to be a purely epiphenomenal property of human action; one can easily see how this produces more efficient, smoother speech, and a more careful use of the human speech-production “hardware.” An analogous action blending effect occurs when people reach for objects: people adaptively flex their fingers while moving the hand toward an object (Jeannerod et al., 1995), and has been observed to develop when sequentially moving a cursor through a learned series of stimuli (Kachergis et al., under review). Compared to typical step-wise robotic motion, this action blending seems to be more efficient, using predictive motion to minimize the time and energy required to achieve the goal.

Further insights into human sequential action planning come from Gentner et al. (1980), who conducted a photographic study of a skilled typist. Using high-speed photography, they analyzed the hand movements of a 90-wpm typist, and found that the typist’s hands were moving continuously, with fingers starting to move toward a destination before several preceding characters were to be typed. In fact, for 96% of all keystrokes, movement was initiated on average 137 ms before the preceding keystroke was completed, and for 21% the movement was initiated before the preceding keystroke was initiated. Larochelle (1984) presents a similar but more extensive study, analyzing the typing of four professional typists while they typed either words or non-words, of which half were typed with one hand, and the other half with two hands. In more than half of the trials the movement was initiated before completion of the previous keystroke for two-handed trials.

These interactions between early and later sequence elements cast doubt on a simple chaining theory of sequential action. Rosenbaum et al. (2007) interpreted these findings as evidence that sensory feedback is not a necessary component for action sequencing, in keeping with the conclusion of Lashley (1951). They argued that “the state of the nervous system can predispose the actor to behave in particular ways in the future,” (p. 526), or, there are *action plans* for some behaviors. And yet, studies on spontaneous speech repair (e.g., Nakatani and Hirschberg, 1994) also show that people are very fast in fixing errors in early components of a word or sentence, much too fast to assume that action outcomes are evaluated only after entire sequences are completed. This means that action planning cannot be exclusively feedforward, as Lashley (1951) seemed to suggest, but must include several layers of processing, with lower levels continuously checking whether the current action component proceeds as expected. In other words, action planning must be a temporally extended process in which abstract representations to some extent provide abstract goal descriptions, which must be integrated with lower-level subsymbolic representations controlling sensorimotor loops. The existence of subsymbolic sensorimotor representations would account for context and anticipation effects, as described above. In the more general field of knowledge representation, some authors even take it one step further, positing that subsymbolic, sensorimotor representations are *necessary* for higher-level symbolic cognition. For example, Barsalou’s (1993, 1999) perceptual symbol systems theory defines cognition as embedded in the world, stating that agents form grounded models via perception and interaction with their environments. With these models, the representation of abstract concepts can be implemented using grounded perceptual symbols. The empirical support for theories like these motivate the notion that both symbolic and subsymbolic representations can (and should) work together to account for human cognition.

A good example for an action planning model that includes one symbolic and one subsymbolic level is the typewriting model suggested by Rumelhart and Norman (1982). To control typing the word “WORD,” say, the model would assume that the symbolic/“semantic” representation WORD would activate motor units controlling the finger movements required to type “W,” “O,” “R,” and “D” in parallel. This parallel activation allows for crosstalk between the different units, which would account for context effects and anticipations. At the same time, the activated units are prevented from firing prematurely by means of a forward-inhibition structure. That is, each unit is inhibiting all following units in the sequence (so that the “W” unit inhibits the “O,” “R,” and “D” units, the “O” unit the “R” and “D” units, and the “R” the “D” unit) and release that inhibition only once they are executed. The dynamics of these inhibition and release processes automatically produce the necessary sequence. It is thought that such activation and inhibition processes play a role even in young infants (Verschoor et al., unpublished). Immediate feedback, though not explicitly addressed by Rumelhart and Norman (1982), could serve to repair the actions controlled by particular units, but the feedback would not be needed to produce the sequence – a major advantage over chaining models. For an overview of similar models and other action domains, see Logan and Crump (2011).

The main lesson for robotic everyday action control is that purely symbolic planning may be too crude and context-insensitive to allow for smooth and efficient multi-component actions. Introducing multiple levels of action planning and action control may complicate the engineering considerably, but it is also likely to make robot action more flexible and robust – and less “robotic” to the eye of the user.

## INTEGRATING FEEDFORWARD AND FEEDBACK MECHANISMS

In perfectly predictable environments such as industrial construction halls, there is hardly any need for feedback mechanisms. Indeed, early industrial robots, such as Unimate, could rely on fully preprogrammed feedforward control for repetitive multi-component actions such as picking up and manipulating objects (Hägele et al., 2008). However, real-life environments are much too unpredictable to allow for purely feedforward control. Considering that purely feedback-based control is often much too slow to allow for real-life human action, it is unsurprising that human action control seeks for an optimal integration of feedforward and feedback mechanisms.

One of the earliest studies into feedforward planning is Henry and Rogers (1960), which compared reaction times of participants performing a simple finger movement to reaction times of a moderately complex arm movement (reaching and grasping) in response to a stimulus. The authors found that participants performing the more complex movement showed a 20% increase in reaction time, with as much as a 25% increase for even more complex movement. This suggests the existence of feedforward action planning prior to action execution.

Linguistic studies have shown a similar effect. Eriksen et al. (1970) had participants read aloud two-digit numbers consisting of a varying number of syllables. Longer numbers were shown to have a longer onset delay. In order to account for the possibility that factors other than motor planning play a role, participants were given the same task with a delay between stimulus onset and vocalization. Here, the effect disappeared, again providing evidence for pre-execution action plan formation.

However, while it may be tempting to conclude that an action plan is formed completely before action onset, incremental approaches to sequential action posit that this is not the case. Palmer and Pfordresher (2003) argued that it is unlikely for actors to have access to all elements in a long sequence, as this would place unnecessarily large demands on memory – just think of a conductor starting to conduct a 4-h Wagner opera. Instead, planning and execution co-occur in time, limiting access to sequence elements that appeared much earlier or that lie far in the future. Evidence for this was indeed found by Sternberg et al. (1988), in which six participants prepared and produced sequences of mono- or tri-syllabic words. In addition to the length effect discussed above, preparation times were found to increase with length of the word sequence until approaching asymptote (which was 10.3 ± 0.6 words for sequences of mono-syllabic words and 6.4 ± 0.9 words for tri-syllabic words). This suggests that plan formation and execution occur simultaneously, at least for longer sequences of actions, with a limited capacity.

However, feedforward mechanisms alone cannot account for such complex action as our tea-making example. A complete feedforward program would need to incorporate numerous unknown parameters, such as the exact location and physical properties (e.g., weight) of all necessary objects. The prior unavailability of such parameters is not the only reason feedback mechanisms might be helpful. Some parameters might be possible to include in a feedforward program, but would simply be more efficient or optimal if filled in online, such as grip strength. Even if all this information were available, an actor still needs to be able to correct possible – sometimes inevitable – perturbations in action execution.

Indeed, it seems that the presence of uncertainty (i.e., unavailability of necessary parameters) increases the importance of feedback mechanisms. Saunders and Vijayakumar (2011) fitted participants with a prosthetic hand that could provide vibrotactile feedback. Using this prosthetic hand, they were asked to manipulate objects of different weights. Manipulating both feedforward uncertainty by adding an unpredictable delay in the prosthetic hand and feedback information by manipulating vibrotactile feedback, they found that performance decreased when feedback was removed in situations with feedforward uncertainty. This illustrates that human action emerges from the interaction of feedforward and feedback mechanisms.

Integrating feedforward and feedback mechanisms holds the promise to get the best from two worlds. Feedforward mechanisms are likely to determine the necessary action components and pre-load at least some of them before initiating the action (Henry and Rogers, 1960), and to selectively tune attention to stimuli and stimulus dimensions that are relevant for the task (Hommel, 2010). Feedback processes, in turn, provide excellent accuracy – often at the cost of speed (Seidler et al., 2004). These strengths and weaknesses have motivated hybrid models claiming that feedforward mechanisms provide the skeleton of action plans which leave open slots for parameters provided by feedback processes (Schmidt, 1975; Glover, 2004; Hommel, 2010). A particularly good example of this kind of interaction is provided by the observations of Goodale et al. (1986). In a clever experiment, participants were asked to rest their hand on a platform and point to a visual target presented at a random location on an imaginary line in their right visual field. The participants were not told that in half of the trials the target changed location during the first saccade. The authors found that participants would successfully point to the target on these trials without even being aware of the location change, and without additional delay. As feedforward programming is thought to take time, a fast and online feedback mechanism of which participants are unaware has to be responsible for this finding. After this study showing online adaptation of hand velocity, Prablanc and Martin (1992) found that these results generalize to two dimensions. Using stimuli presented on a screen, it was found that both the velocity and trajectory of the hand were adjusted online. This demonstrates that action is the result of a preprogrammed action plan (the initial movement of the hand) combined with online adaptation to reach goal requirements. Interestingly, such a division of labor fits well with the architecture of the human brain, which includes both a slow, cognitively penetrated ventral route from perception to action and a fast dorsal sensorimotor loop (for a broader overview, see Milner and Goodale, 1995).

It is clear that both feedforward and feedback mechanisms are responsible for producing complex action, but there remain a number of unanswered questions. Are feedforward processes always responsible for certain actions? How are these plans learned, and how do people know when to apply them? How does feedback on a lower level result in action re-planning on a higher level, and does this require conscious intervention? What is the division of labor between feedback and feedforward mechanisms? How fluid is it – how hierarchical?

We know that with practice, the roles of feedback and feedforward processes change. In a standard rapid aimed limb movement paradigm, participants are asked to perform a manual action in order to reach a target. During such tasks, the response can be regarded as having two elements: (1) a ballistic primary movement, thought to be controlled by a feedforward mechanism, and (2) a secondary, corrective movement, thought to be caused by a feedback mechanism. Pratt and Abrams (1996) used such a paradigm to investigate the effect of practice on the weight of primary and secondary movements. Participants were asked to repeatedly move a visual cursor to a target location using wrist rotation. With more practice, the percentage of time spent in the first movement increased, while time spent in the second movement decreased. As the first movement is feedforward-controlled, this suggests that practice reduces the need of feedback control, as the feedforward process becomes more accurate. But will this learning generalize to new situations with similar action requirements, and is it long-lasting?

To investigate the relationship between practice and feedback control, Proteau et al. (1987) had participants practice an aiming task on either 200 or 2000 trials and found that, when visual feedback was taken away, participants who had more practice were more impaired by the removal of feedback. This is not what one would expect if practice simply shifts control to feedforward processes. Subsequent research has shown that, with practice, higher peak velocities are reached in the early phase of movement, thereby leaving more time for corrective submovements based on feedback. Thus, instead of a shift from feedback control to feedforward control, feedback processes seem to be optimized as a result of practice (Proteau et al., 1987; Khan et al., 1998; Elliott et al., 2010).

While the first generation of robots and other intelligent systems had a strong preference for feedforward control, not in the least because of the rather predictable environments they were implemented in, some modern systems rely heavily on feedback control to perform actions – especially humanoid systems operating in real-world scenarios. This is likely to work as long as action production in such robots is slower than the feedback loops informing them (Plooij et al., 2013), but progress in action mechanics is likely to make hybrid feedforward/feedback systems an attractive alternative in the near future.

## HIERARCHICAL ACTION REPRESENTATION

Human actions can often be described in a hierarchical fashion: “Going on vacation” implies action such as “packing my bags,” “getting the car,” “loading it,” “driving down to city X,” and so forth and so on. Many authors have taken that to imply that action control is hierarchical as well. According to Lashley (1951), only a hierarchical organization of actions and action plans can provide the opportunity to have the same motor acts acquire different meanings, depending on the context in which the motor act is performed. In Miller et al. (1960) seminal book, action plans are even hierarchical by definition: “A Plan is any hierarchical process in the organism that can control the order in which a sequence of operations is to be performed” (p. 16). And yet, while it is certainly uncontroversial that it is possible to *describe* actions as hierarchical, this need not have any implication for the cognitive organization of actions. As Badre (2008) argues, “the fact that a task can be represented hierarchically does not require that the action system itself consist of structurally distinct processing levels” (p. 193; see also Klein, 1983). Moreover, it is not always clear what authors mean if they say that actions are organized in a hierarchical fashion.

Uithol et al. (2012) noted that there are at least two ways to look at hierarchical action. These two ways differ in what are considered to be the different levels in such a hierarchy. One way to look at action hierarchies is the view of part-whole relations. In this account, each level in the hierarchy exists solely as the sum of lower-level units. In other words, an action unit such as “get a pan for pancake making” consists of the subunits “open the cupboard,” “take pan from cupboard,” “place pan on counter,” and “close the cupboard.” It should be clear that when all subordinate units are present, the superordinate unit “get a pan” is also present, as it is identical to the sum of its parts. Uithol et al. (2012) argues that this kind of hierarchy does not provide an explanation of the complex action; it merely provides a thorough description of the to-be-explained action, in which higher levels are more complex than lower levels. It also does not give information about the causal relationship between the different levels in the hierarchy, as you cannot consider an element to be the cause of its own parts. Another restriction of this type of hierarchy is that it can only accommodate levels that are of a similar nature. That is, actions can only be divided into sub-actions, not into objects or world states.

Another way to view hierarchies is to see the different levels as representing causal relations between the levels. In this approach, units on a higher level causally influence units on a lower level. In this type of hierarchy, lower-level units can be modulated by higher-level units. In contrast with the part-whole hierarchy, lower levels are not necessarily less complex than higher levels. Goals that are formulated as simple and propositional states can be the cause of more complex elements. Using this hierarchical approach also opens up the possibility of states or objects being the cause of an action, as it does not have the limitation of requiring action-type goals.

Uithol et al. (2012) proposed a new model, in which the fundamental foundation for the hierarchical structure is not cause-and-effect (i.e., goals cause motor acts), or complexity (i.e., complex motor acts such as grabbing a pan consist of simpler acts such as flexing fingers and grasping the handle), but temporal stability. In this view, stable representations can be considered goal-related, while more temporary representations reflect motor acts on different levels, not unlike the more enduring conceptual representations and the less enduring motor units of Rumelhart and Norman’s (1982) model discussed above. However, this representation proposal does not include a model of how the hierarchies within a task are abstracted and learned from experience, nor of how they may be shared across tasks despite requiring different parameterizations.

Botvinick and Plaut (2004) tackled some of these issues, pointing out that not only is it unclear how existing hierarchical models learn hierarchies from experience, but also that most theoretical accounts lead to a circular reference: acquiring sequence knowledge relies on the ability to identify event boundaries, which in turn requires sequence knowledge. A further problem is sequencing in hierarchical structures; many models (e.g., Rumelhart and Norman, 1982; Houghton, 1990) solve that by means of forward**inhibition, but this only works on units at the lowest level of a hierarchy. Botvinick and Plaut (2004) offered a recurrent connectionist network model that helps avoiding these problems. Using computer simulations they showed that such a network, which contains no inherent hierarchical structure, can learn a range of sequential actions that many consider hierarchical. The hierarchy, they argued, emerges from the system as a whole. The network they used is a three-layer recurrent network, with an input layer representing held objects and fixated objects, an output layer representing actions to be taken, and a hidden layer (with recurrent connections) for the internal representation. Having trained this network on a routine complex task (making coffee or tea), they showed that it can perform complex action that can be considered hierarchical in nature (e.g., varying orders of subactions leading to the same outcome) without relying on a hierarchical system architecture. The network also showed slips of action when the internal representation layer was degraded, as well as other action errors found in empirical studies, although Cooper and Shallice (2006) suggest that the relative frequency and types of errors shown by the recurrent model do not match human subjects.

We believe that architectures offering such hierarchical behavior, without necessarily being hierarchically structured, can provide robots with the needed flexibility to function in a dynamic, human-driven world. Botvinick and Plaut’s (2004) model seems to be able to account for some aspects of flexible behavior, but more complex and biologically inspired models such as LEABRA (O’Reilly, 1996; Kachergis et al., under review) promise to generalize to other tasks, as well as being able to learn relatively fast, two aspects of human behavior we consider essential to emulate in robot behavior.

## CONTEXTUALIZING ACTION CONTROL

As pointed out above, one of the reasons why Lashley (1951) considered action representations to be necessarily hierarchically organized was the fact that the meaning and purpose of action components vary with the goal that they serve to accomplish: while making a kicking movement with your right leg can easily be replaced by moving your head sideways when trying to score a goal in a soccer game, that would not be a particularly good idea when performing a group can-can on stage during a performance of Orpheus in the Underworld. In other words, goals are needed to contextualize action components. In AI, robotics, and some information-processing approaches in psychology, the main function of goal representation is to guide the selection of task components, including stimulus and response representations or perception-action rules. In traditional processing models, like ACT-R or Soar (Laird et al., 1987; Anderson, 1993), goal representations limit the number of production rules considered for a task, which reduces the search space and makes task preparation more efficient (Cooper and Shallice, 2006). Moreover, goals commonly serve as a reference in evaluating an action, when comparing the current state of the environment with the desired state (Miller et al., 1960).

This practice was challenged by Botvinick and Plaut (2004), who pointed out at least two problems with goal representations in cognitive models. First, goals themselves may be context-dependent. The goal of cleaning the house may have rather different implications depending on whether it serves to satisfy the expectations of one’s partner or to prepare for a visit of one’s mother-in-law. Likewise, the goal of stirring will produce somewhat different behavior depending on whether one is stirring egg yolks or cement. Most models that postulate the existence of goals do not allow for such context dependence. Second, it is argued that many everyday activities do not seem to have definable, or at least not invariant goals; just think of playing a musical instrument or taking a walk. The authors demonstrated that goal-directed behavior can be achieved without the explicit representation of goals. In the previously mentioned simulation studies with recurrent neural networks, they were able to simulate goal-directed actions that operate very much like Miller et al.’s (1960) TOTE units, without any need to represent the goal explicitly. Obviating the need for representing goals, such a model could be applied to behavior with non-obvious goals, such as taking a walk as a consequence of feeling restless or having the thought of fresh air (Botvinick and Plaut, 2004).

Cooper and Shallice (2006) took issue with this non-representationalist account of goals, giving at least two reasons why goals *should *be implemented in cognitive models. First, goals allow for the distinction between critical and supporting actions. When making pancakes, the subaction of adding egg to the mixture consists of picking up an egg, breaking it (above the bowl), and discarding the empty shell (not above the bowl). It should be clear that the breaking of the egg is the most important action in this sequence. Dissociating important actions from non-important actions can account for skipping unnecessary steps. When applying butter to two slices of toast, it is not necessary to execute the supporting actions “discard knife” and “pick up knife” between the two executions of the “butter toast” action program. Second, the implementation of goals would allow for subactions that serve the same purpose to be interchanged. For example, flipping a pancake by flipping it in the air or flipping it using a spatula would both be perfectly good methods for pancake flipping, and the shared goal allows these actions to be interchanged. Models without goal representation can only show this behavior if they are explicitly trained on all the alternative actions that can be taken. To make the realization that a set of actions are equivalent for achieving a goal, a model would in essence have to contain a representation of that goal.

Interestingly, however, goal representations (whether explicit or implicit) can play an important role in contextualizing cognitive representations. Most representational accounts assume that representations of stimulus and action events are invariant. The need to contextualize representations – i.e., to tailor them to the particular situation and task at hand – thus seems to put the entire burden on the goal, so that the explicit representation of the goal seems to be a necessary precondition for adaptive behavior. But, from a grounded cognition perspective, it seems that alternative scenarios are possible. In a grounded cognition framework, the representation of objects and object categories takes an embodied form, using modal features from at least the visual, motor, and auditory modalities (Prinz and Barsalou, 2000). For example, the concept of apple would be represented by a network of visual codes representing <green> and <round>, but also the auditory <crunchy sound> of biting into it. The embodied cognition framework has already been successfully implemented in robot platforms such as iCub, and shows stimulus compatibility effects similar to those that can be observed in humans (Macura et al., 2009; Pezzulo et al., 2011).

According to the Theory of Event Coding (Hommel et al., 2001), events are represented – like objects – in a feature-based, distributed fashion. This will mean that the aforementioned apple would be represented by a network of codes representing not only the apple’s perceptual features such as being <greenish> and <round>, but also its properties such as being <edible>, <graspable>, <carryable>, <throwable>, and so forth. In this view, one of the main roles of goals is to emphasize (i.e., increase the weight of) those features that in the present task are of particular importance. This means that when hungry, the feature of being <edible> will be primed in advance and become more activated when facing an apple, while <throwability> will become more important when being in danger and trying to defend oneself. Several studies have provided evidence that goals are indeed biasing attentional settings toward action-relevant feature dimensions (e.g., Fagioli et al., 2007; Wykowska et al., 2009; Kühn et al., 2011), suggesting that the impact of goals goes beyond the selection of production rules and outcome evaluation. Interestingly, this kind of “intentional weighting” function (Memelink and Hommel, 2013) can be considered to represent the current goal without requiring any explicit representation – very much along the lines of Botvinick and Plaut (2004).

Another potential role of goals is related to temporal order. In chaining models, the dimension of time was unnecessary because the completion of each component automatically “ignites” the next component. The same holds for current planners in cognitive robotics, which commonly fix the order of action subcomponents (e.g., CRAM: Beetz et al., 2010). But action plans may follow a more abstract syntax instead, much like how syntactic constraints of natural languages allow for various possible sequences. For instance, consider the process of making tea. With the possible exception of true connoisseurs, it doesn’t make any difference for most tea drinkers whether one puts the tea or the water into the cup first; i.e., the order of these two subactions is interchangeable. A truly flexible system would thus allow for any of these orders, depending on whether water or tea is immediately at hand. While a chaining model would not allow for changing the original order, a more syntactic action plan would merely define possible slots for particular subcomponents (e.g., Rosenbaum et al., 1986), so that the actual order of execution would be an emerging property of the interaction of the syntactic plan and the situational availability of the necessary ingredients.

These considerations suggest that robotic systems need to incorporate at least some rudimentary aspects of time and temporal order to get on par with humans. Along these lines, Maniadakis and Trahanias (2011) have propagated the idea that robotic systems should be equipped with some kind of temporal cognition, be it by incorporating temporal logic or event calculus. Indeed, recent robotic knowledge representation systems, such as KnowRob (Tenorth and Beetz, 2012), do possess the ability to do spatiotemporal reasoning about the changing locations of objects, such as predicting when and where objects can be found.

## CONCLUSION

We have discussed how conceptions of robotic action planning can benefit from insights into human action planning. Indeed, we believe that constructing truly flexible and autonomous robots requires inspiration from human cognition. We focused on four basic principles that characterize human action planning, and we have argued that taking these principles on board will help to make artificial cognition more human-like.

First, we have discussed evidence that human action planning emerges from the integration of a rather abstract, perhaps symbolic representational level and concurrent planning at a lower, more concrete representational level. It is certainly true that multi-level planning can create difficult coordination problems. Using grounded cognition approaches in robotics is potentially a good method to ground such higher-level symbolic representation in lower-level sensorimotor representations, which may allow robot action to become more flexible and efficient.

Second, we have argued that human action planning emerges from the interplay of feedforward and feedback mechanisms. Again, purely feedforward or purely feedback architectures are likely to be more transparent and easier to control. However, fast, real-time robotic action in uncertain environments will require a hybrid approach that distributes labor much like the human brain does by combining slow and highly optimized feedforward control with fast sensorimotor loops that continuously update the available environmental information. A major challenge for the near future will be to combine such hybrid systems with error-monitoring and error-correcting mechanisms. When preparing pancake dough, accidentally pouring some milk outside the bowl would need to trigger a fast correction mechanism informed by low-level sensory feedback but not necessarily the re-planning of (or crying over) the entire action. However, if for some reason the entire milk carton is emptied by this accident, leaving the agent without the necessary ingredient, feedback would have to propagate to higher, more abstract or more comprehensive planning levels to decide whether the plan needs to be aborted. How this works in detail and how decisions are made as to which level is to be informed is not well understood, but progress is being made. Research into feedback processes has yielded information about the optimal speed of sensorimotor loops (Joshi and Maass, 2005), and we find it reasonable to expect that models using such fast feedback loops combined with accurate feedforward planning can ultimately produce human-like motor performance in robots.

Third, we have argued that while descriptions of human actions may refer to a hierarchy, it is not yet clear whether the cognitive – *in vivo* or *in silico* – representations of such actions need to be explicitly hierarchical as well. Equally unclear is whether representations that differ in hierarchical level would necessarily need to differ in format. However, it is clear that representations that are considered to be “higher in hierarchy” are more comprehensive. The concept of “making a pancake,” say, is necessarily richer and more abstract than the associated lower-level actions of “reaching for egg” and “grabbing a pan,” suggesting that the latter two are more directly grounded in sensorimotor activity (Kraft et al., 2008). Future research will need to investigate how representations at different planning levels (or different levels of description) interact or relate to each other.

The nature of goals and their role in action control is also a matter of ongoing research. The two different viewpoints – i.e., that goals require explicit representation or not – seem to reflect different preferences in conceptualization and modeling techniques, and it may well turn out that an explicit representation of goals in the preferred modeling language translates to a more implicit representation of goals in the actual functional or neural architecture. In robotics, most modern plan languages use a form of explicit goal-related action control that defines a goal as a required world state on which constraints can be imposed. Such a structure is flexible enough to allow equifinality, but it is unclear how knowledge about the various means to produce a result is acquired. Ultimately, we believe that subsymbolic programming approaches may allow for more adaptive, “human” representational architectures – though likely more difficult to engineer and define provably safe operating conditions for.

To conclude, we believe that the construction of robots that are up to real-life, everyday actions in environments that are as uncertain as human environments requires the consideration of cognitive principles like the four principles we have discussed in this article. The benefit of doing so will be twofold. For one, it will strongly increase the flexibility of robots. For another, it will make robots more human-like in the eyes of the human user, which will help us understand and cooperate with our future robotic colleagues.

## Conflict of Interest Statement

The authors declare that the research was conducted in the absence of any commercial or financial relationships that could be construed as a potential conflict of interest.
